# Scientific Items

**Published:** 1884-05-20

**Authors:** 


### SCIENTIFIC ITEMS.

**Mending a Submarine Cable.**—When a submarine telegraphic cable is accidentally broken, the distance of the break from shore can be determined with great precision by electric tests; but those who are not familiar with the business naturally think that fishing up the broken thread in the midst of the ocean must be a feat equal to the proverbial "finding a needle in a haymow." The ease and certainty with which the thing is done is, indeed, amazing; but we get used to scientific wonders nowadays. Some time ago one of the lines of the Anglo-American Company was caught without trouble, at a depth of two miles and a quarter, near the middle of the Atlantic.

The machinery used for picking up a cable consists of a rope, about an inch and a quarter in diameter, made from the strongest hemp with interwoven wires of fine steel. The grapnel at the end is merely a solid shaft of iron some two feet long, and weighing about a hundred pounds, and prolonged into six blunt hooks, which much resemble the partly closed fingers of the human hand.

In picking up the cable in deep water, the vess.l, after reaching the waters near the break, lets out her rope and grapnel, then takes a course at right angles to the cable, and at some distance from the fracture. so that the broken ends may not slip through the grapnel. The grapnel-rope is attached to a dynamometer, which exactly measures the strain on the rope, and shows unerringly when the cable has been caught. If the grapnel fouls a rock, the strain rises very suddenly to a high point; but, the exact weight of the cable being known, the dynamometer signals by the steady rate of increase its hold on the cable far below.

The splicing of the broken cable is a work of great delicacy and skill, but, when accomplished by trained fingers, the spliced part can scarcely be distinguished from the main cord —*Pop. Sci. News.*

**The Achromatic Microscope.**—Oliver Wendell Holmes, in an address to the Harvard Medical School, referred to the achromatic microscope as having "created a new era in medical science," to say nothing of its great services in other departments of knowledge. He illustrated the power of the instrument strikingly by saying, while a scrap of human skin was under the glass, that the fragment thus magnified represented an individual just one mile in height. He would ten times overtop the loftiest of the pyramids, and twenty times the tallest of our steeples. His breadth and thickness being in proportion to his height, his weight would be one hundred and twenty billion pounds, equal to sixty million tons. "He could take our State House up as we would lift a paving-stone," the Doctor added, "and fling it into the waters beyond Boston Lighthouse."—*Ibid.*

**Carbonic Acid and the Devil.**—When Friedrich Hoffman discovered carbonic-acid gas, and traced its effects on animal life, he was denounced by more than one German university as hostile to religion, and verging toward atheism. Three or four students at the University of Jena, in the attempt to raise a spirit for the discovery of a supposed hidden treasure, were strangled or poisoned by the fumes of the charcoal they had been burning in a close garden-house of a vineyard near Jena, while employed in their magic fumigations and charms. Only one was restored to life, and, from his account of the noises and spectres in his ears and eyes as he was losing his senses, it was taken for granted that the bad spirit had destroyed them. Hoffmann admitted that it was a very bad spirit that had tempted them, the spirit of avarice and folly; and that a very noxious spirit—gas, or *geist*—was the immediate cause of their death. But he contended that this latter spirit was the spirit of charcoal, which would have produced the same effect had the young men been chanting psalms instead of incantations, and acquitted the Devil of all direct concern in the business. The theological faculty took alarm; even physicians pretended to be horror-stricken at such audacity.

**Pressure in a Diving-bell.**—A diving-bell allows us to perceive a sudden increase of pressure, but not by the ordinary sense of touch. The hand does not perceive the difference between fifteen pounds per square inch, pressing it all around, and seventeen pounds, or eighteen pounds, or twenty pounds, or even thirty pounds per square inch, as is experienced when you go down in a diving-bell. If you go down five-and-a-half fathoms in a diving-bell, your hand is pressed all around with a force of thirty pounds to the square inch, but yet you do not perceive any difference in the sense of force, any perception of pressure. What you do perceive is this: Behind the tympanum is a certain cavity filled with air, and a greater pressure on one side of the tympanum than on the other gives rise to a painful sensation, and sometimes produces rupture of it, in a person going down in a diving-bell suddenly. The remedy for the painful sensation thus experienced, or rather I should say its prevention, is to keep chewing a piece of hard biscuit, or make believe to do so. If you are chewing a hard biscuit, the operation keeps open a certain passage, by which the air-pressure gets access to the inside of the tympanum and balances the outside pressure, and thus prevents the painful effect. This painful effect on the ear, experienced by going down in a diving-bell, is simply because a certain piece of tissue is being pressed more on one side than on the other; and when we get such a tremendous force on a delicate thing like the tympanum we may experience a great deal of pain, and it may be dangerous; indeed, it is dangerous, and produces rupture or damage to the tympanum unless means be adopted for obviating the difference in the pressure; but the simple means I have indicated are, I believe, with all ordinary healthy persons, perfectly successful.—*Nature.*

**Subscribe** for the SOUTHERN MEDICAL RECORD.

